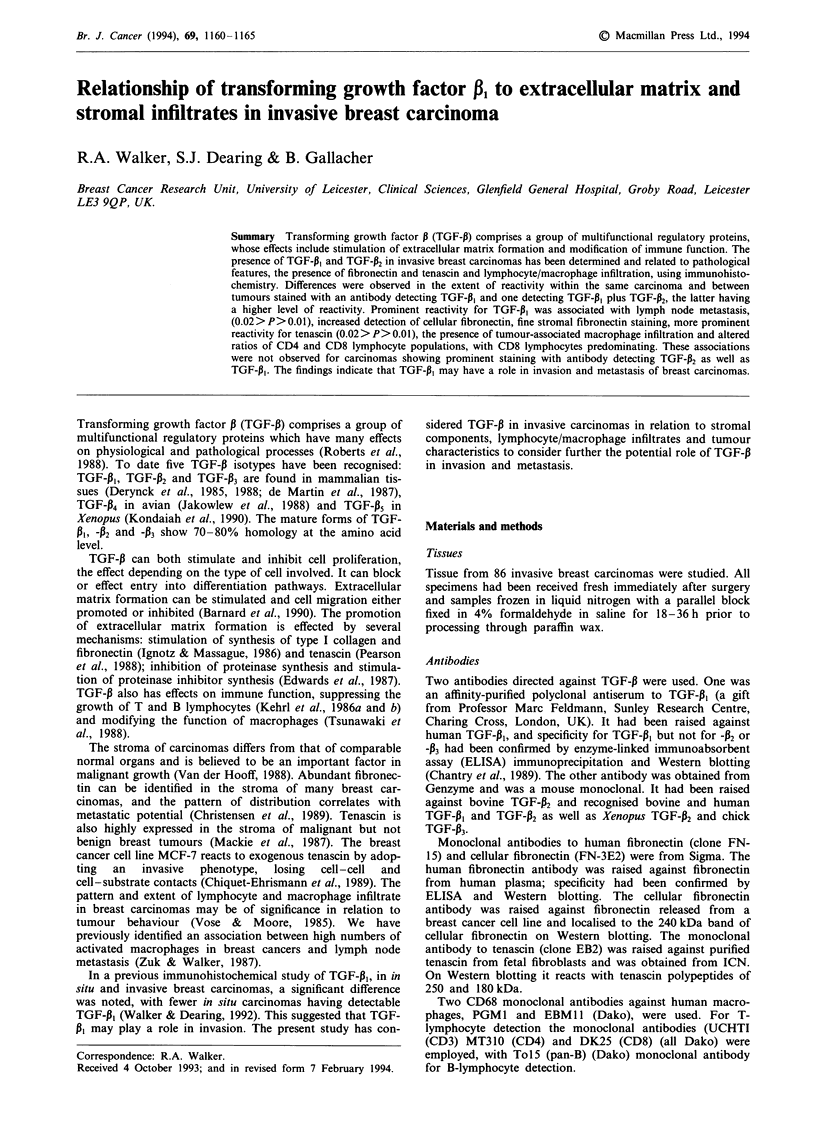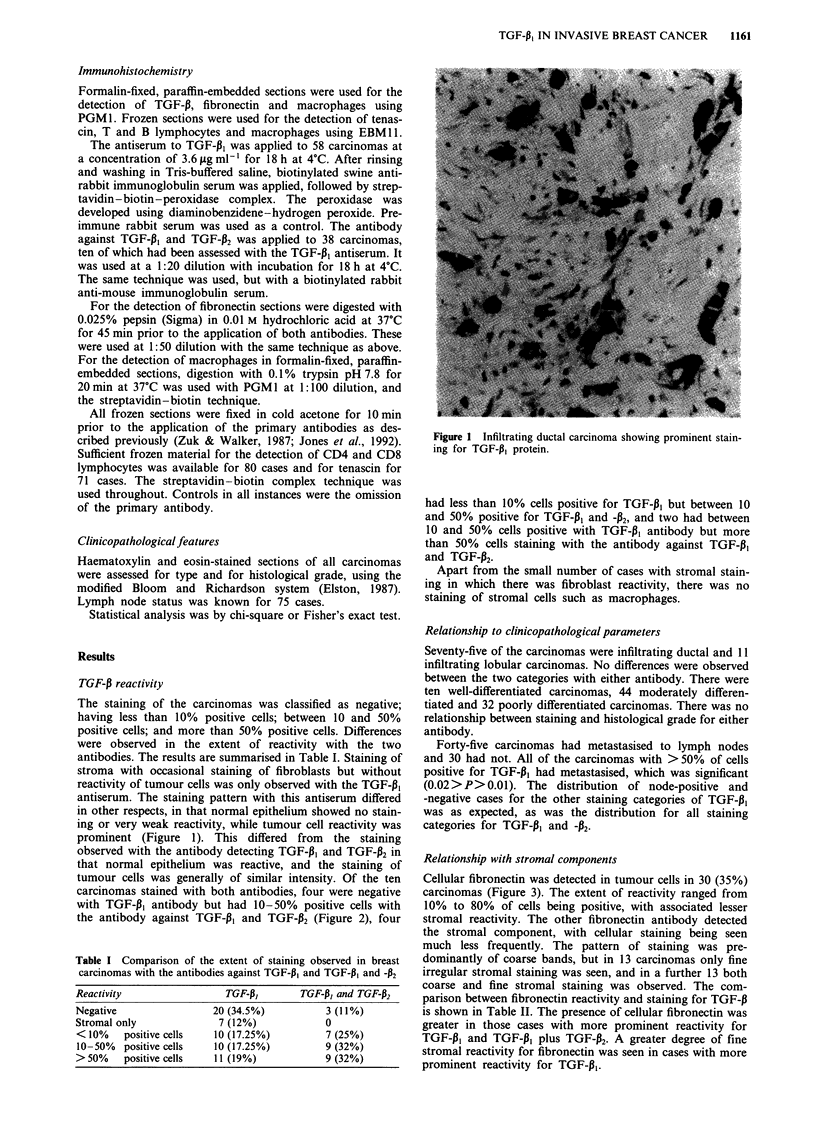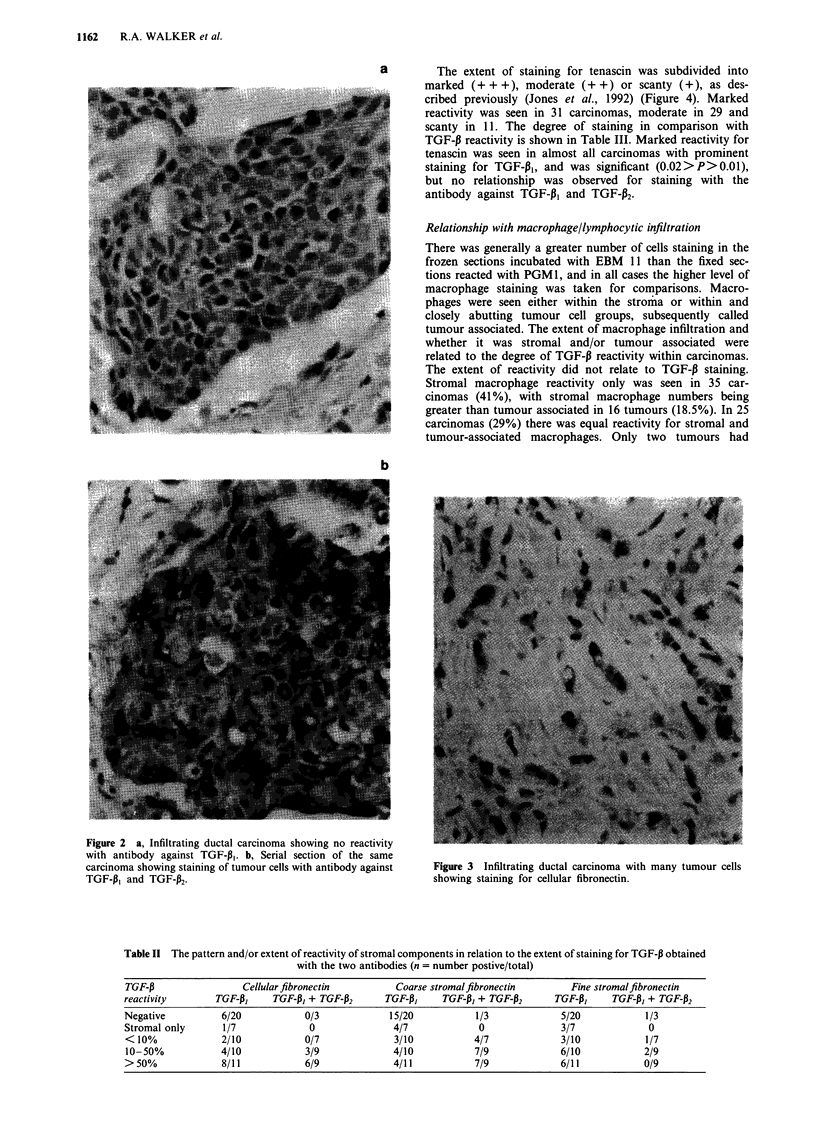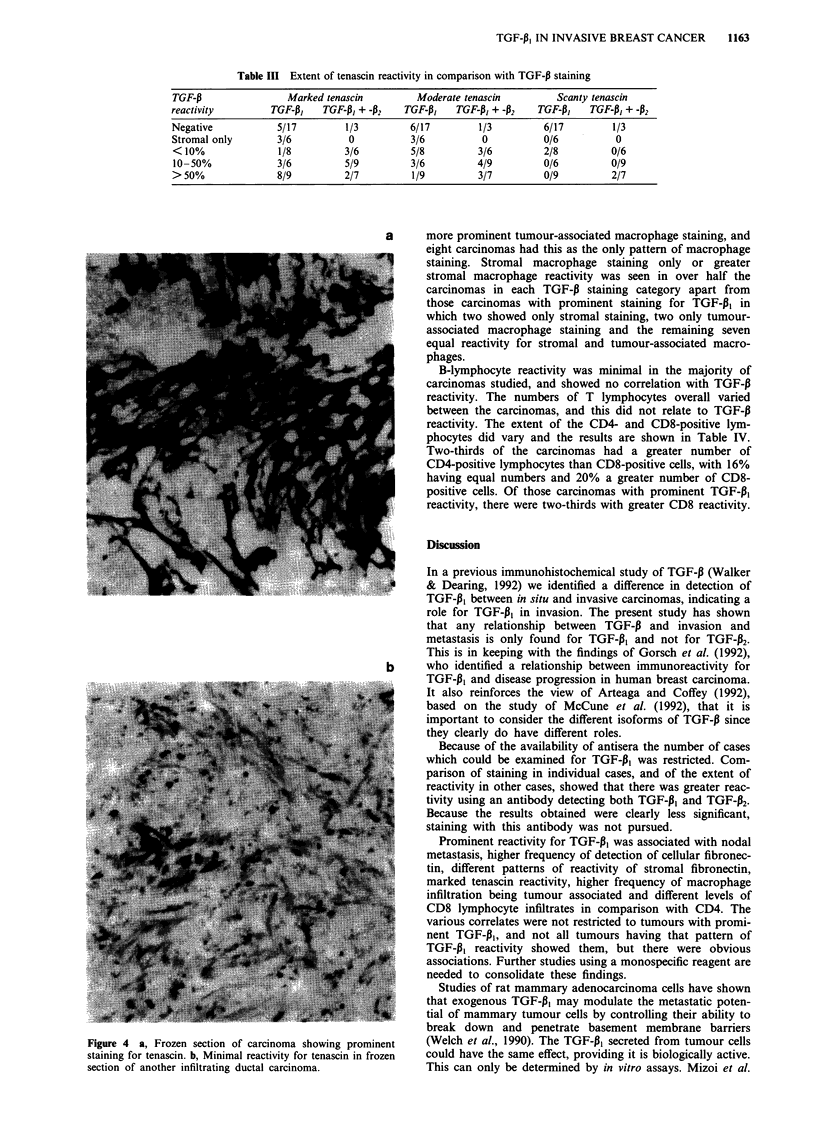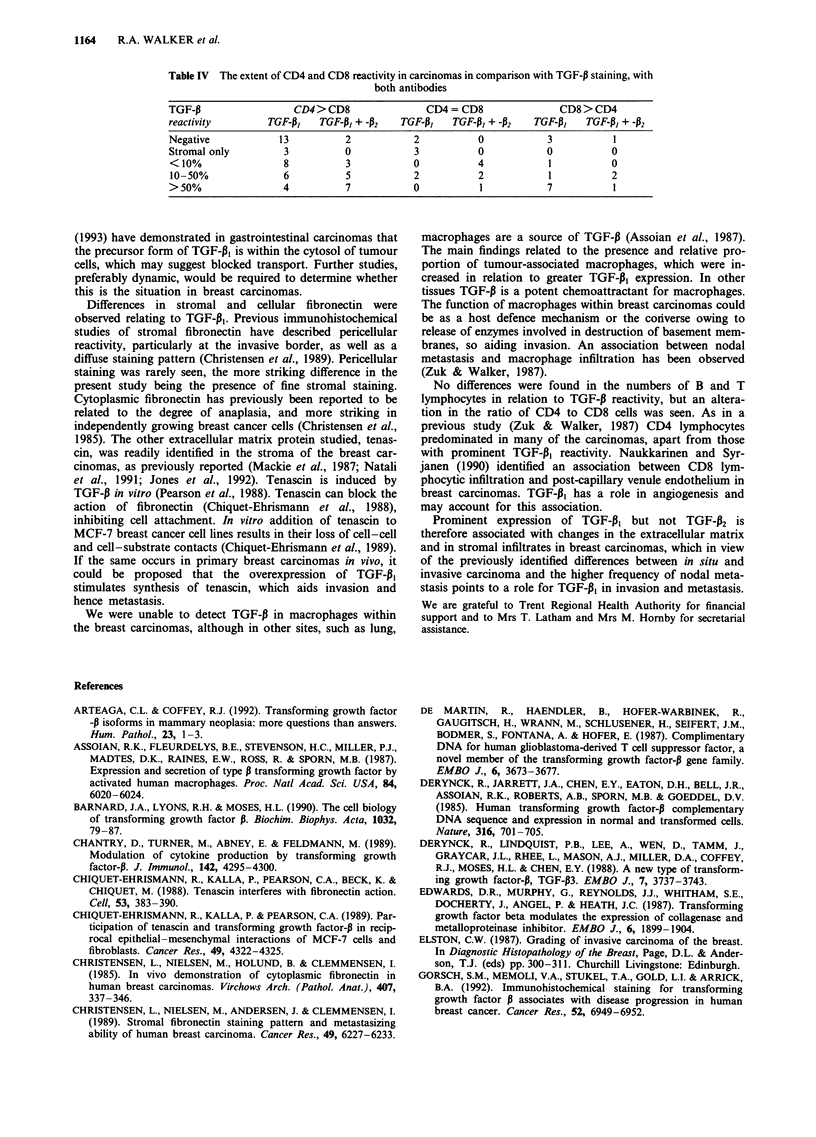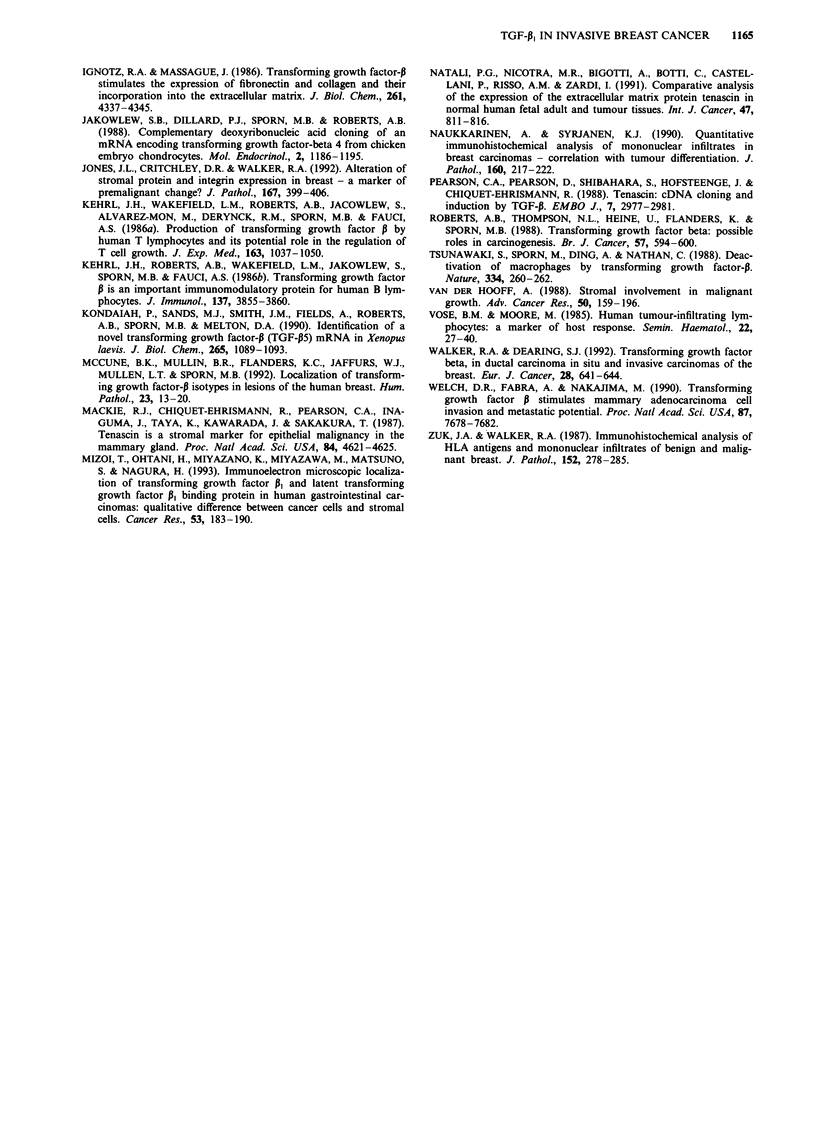# Relationship of transforming growth factor beta 1 to extracellular matrix and stromal infiltrates in invasive breast carcinoma.

**DOI:** 10.1038/bjc.1994.228

**Published:** 1994-06

**Authors:** R. A. Walker, S. J. Dearing, B. Gallacher

**Affiliations:** Breast Cancer Research Unit, University of Leicester, Glenfield General Hospital, UK.

## Abstract

**Images:**


Br. J. Cancer (1994), 69, 1160-1165 © Macmillan Press Ltd., 1994

### Relationship of transforming growth factor $\beta_1$ to extracellular matrix and stromal infiltrates in invasive breast carcinoma

R.A. Walker, S.J. Dearing & B. Gallacher

*Breast Cancer Research Unit, University of Leicester, Clinical Sciences, Glenfield General Hospital, Groby Road, Leicester LE3 9QP, UK.*

**Summary** Transforming growth factor $\beta$ (TGF-$\beta$) comprises a group of multifunctional regulatory proteins, whose effects include stimulation of extracellular matrix formation and modification of immune function. The presence of TGF-$\beta_1$ and TGF-$\beta_2$ in invasive breast carcinomas has been determined and related to pathological features, the presence of fibronectin and tenascin and lymphocyte/macrophage infiltration, using immunohistochemistry. Differences were observed in the extent of reactivity within the same carcinoma and between tumours stained with an antibody detecting TGF-$\beta_1$ and one detecting TGF-$\beta_1$ plus TGF-$\beta_2$, the latter having a higher level of reactivity. Prominent reactivity for TGF-$\beta_1$ was associated with lymph node metastasis, ($0.02 > P > 0.01$), increased detection of cellular fibronectin, fine stromal fibronectin staining, more prominent reactivity for tenascin ($0.02 > P > 0.01$), the presence of tumour-associated macrophage infiltration and altered ratios of CD4 and CD8 lymphocyte populations, with CD8 lymphocytes predominating. These associations were not observed for carcinomas showing prominent staining with antibody detecting TGF-$\beta_2$ as well as TGF-$\beta_1$. The findings indicate that TGF-$\beta_1$ may have a role in invasion and metastasis of breast carcinomas.

Transforming growth factor $\beta$ (TGF-$\beta$) comprises a group of multifunctional regulatory proteins which have many effects on physiological and pathological processes (Roberts *et al.*, 1988). To date five TGF-$\beta$ isotypes have been recognised: TGF-$\beta_1$, TGF-$\beta_2$ and TGF-$\beta_3$ are found in mammalian tissues (Derynck *et al.*, 1985, 1988; de Martin *et al.*, 1987), TGF-$\beta_4$ in avian (Jakowlew *et al.*, 1988) and TGF-$\beta_5$ in *Xenopus* (Kondaiah *et al.*, 1990). The mature forms of TGF-$\beta_1$, -$\beta_2$ and -$\beta_3$ show 70–80% homology at the amino acid level.

TGF-$\beta$ can both stimulate and inhibit cell proliferation, the effect depending on the type of cell involved. It can block or effect entry into differentiation pathways. Extracellular matrix formation can be stimulated and cell migration either promoted or inhibited (Barnard *et al.*, 1990). The promotion of extracellular matrix formation is effected by several mechanisms: stimulation of synthesis of type I collagen and fibronectin (Ignotz & Massague, 1986) and tenascin (Pearson *et al.*, 1988); inhibition of proteinase synthesis and stimulation of proteinase inhibitor synthesis (Edwards *et al.*, 1987). TGF-$\beta$ also has effects on immune function, suppressing the growth of T and B lymphocytes (Kehrl *et al.*, 1986*a* and *b*) and modifying the function of macrophages (Tsunawaki *et al.*, 1988).

The stroma of carcinomas differs from that of comparable normal organs and is believed to be an important factor in malignant growth (Van der Hooff, 1988). Abundant fibronectin can be identified in the stroma of many breast carcinomas, and the pattern of distribution correlates with metastatic potential (Christensen *et al.*, 1989). Tenascin is also highly expressed in the stroma of malignant but not benign breast tumours (Mackie *et al.*, 1987). The breast cancer cell line MCF-7 reacts to exogenous tenascin by adopting an invasive phenotype, losing cell–cell and cell–substrate contacts (Chiquet-Ehrismann *et al.*, 1989). The pattern and extent of lymphocyte and macrophage infiltrate in breast carcinomas may be of significance in relation to tumour behaviour (Vose & Moore, 1985). We have previously identified an association between high numbers of activated macrophages in breast cancers and lymph node metastasis (Zuk & Walker, 1987).

In a previous immunohistochemical study of TGF-$\beta_1$, in *in situ* and invasive breast carcinomas, a significant difference was noted, with fewer *in situ* carcinomas having detectable TGF-$\beta_1$ (Walker & Dearing, 1992). This suggested that TGF-$\beta_1$ may play a role in invasion. The present study has considered TGF-$\beta$ in invasive carcinomas in relation to stromal components, lymphocyte/macrophage infiltrates and tumour characteristics to consider further the potential role of TGF-$\beta$ in invasion and metastasis.

Correspondence: R.A. Walker.
Received 4 October 1993; and in revised form 7 February 1994.

### Materials and methods

### *Tissues*

Tissue from 86 invasive breast carcinomas were studied. All specimens had been received fresh immediately after surgery and samples frozen in liquid nitrogen with a parallel block fixed in 4% formaldehyde in saline for 18–36 h prior to processing through paraffin wax.

### *Antibodies*

Two antibodies directed against TGF-$\beta$ were used. One was an affinity-purified polyclonal antiserum to TGF-$\beta_1$ (a gift from Professor Marc Feldmann, Sunley Research Centre, Charing Cross, London, UK). It had been raised against human TGF-$\beta_1$, and specificity for TGF-$\beta_1$ but not for -$\beta_2$ or -$\beta_3$ had been confirmed by enzyme-linked immunoabsorbent assay (ELISA) immunoprecipitation and Western blotting (Chantry *et al.*, 1989). The other antibody was obtained from Genzyme and was a mouse monoclonal. It had been raised against bovine TGF-$\beta_2$ and recognised bovine and human TGF-$\beta_1$ and TGF-$\beta_2$ as well as *Xenopus* TGF-$\beta_2$ and chick TGF-$\beta_3$.

Monoclonal antibodies to human fibronectin (clone FN-15) and cellular fibronectin (FN-3E2) were from Sigma. The human fibronectin antibody was raised against fibronectin from human plasma; specificity had been confirmed by ELISA and Western blotting. The cellular fibronectin antibody was raised against fibronectin released from a breast cancer cell line and localised to the 240 kDa band of cellular fibronectin on Western blotting. The monoclonal antibody to tenascin (clone EB2) was raised against purified tenascin from fetal fibroblasts and was obtained from ICN. On Western blotting it reacts with tenascin polypeptides of 250 and 180 kDa.

Two CD68 monoclonal antibodies against human macrophages, PGM1 and EBM11 (Dako), were used. For T-lymphocyte detection the monoclonal antibodies (UCHTI (CD3) MT310 (CD4) and DK25 (CD8) (all Dako) were employed, with To15 (pan-B) (Dako) monoclonal antibody for B-lymphocyte detection.

*Immunohistochemistry*

Formalin-fixed, paraffin-embedded sections were used for the detection of TGF-$\beta$, fibronectin and macrophages using PGM1. Frozen sections were used for the detection of tenascin, T and B lymphocytes and macrophages using EBM11.

The antiserum to TGF-$\beta_1$ was applied to 58 carcinomas at a concentration of 3.6 $\mu$g ml$^{-1}$ for 18 h at 4°C. After rinsing and washing in Tris-buffered saline, biotinylated swine anti-rabbit immunoglobulin serum was applied, followed by streptavidin–biotin–peroxidase complex. The peroxidase was developed using diaminobenzidene–hydrogen peroxide. Pre-immune rabbit serum was used as a control. The antibody against TGF-$\beta_1$ and TGF-$\beta_2$ was applied to 38 carcinomas, ten of which had been assessed with the TGF-$\beta_1$ antiserum. It was used at a 1:20 dilution with incubation for 18 h at 4°C. The same technique was used, but with a biotinylated rabbit anti-mouse immunoglobulin serum.

For the detection of fibronectin sections were digested with 0.025% pepsin (Sigma) in 0.01 M hydrochloric acid at 37°C for 45 min prior to the application of both antibodies. These were used at 1:50 dilution with the same technique as above. For the detection of macrophages in formalin-fixed, paraffin-embedded sections, digestion with 0.1% trypsin pH 7.8 for 20 min at 37°C was used with PGM1 at 1:100 dilution, and the streptavidin–biotin technique.

All frozen sections were fixed in cold acetone for 10 min prior to the application of the primary antibodies as described previously (Zuk & Walker, 1987; Jones *et al.*, 1992). Sufficient frozen material for the detection of CD4 and CD8 lymphocytes was available for 80 cases and for tenascin for 71 cases. The streptavidin–biotin complex technique was used throughout. Controls in all instances were the omission of the primary antibody.

*Clinicopathological features*

Haematoxylin and eosin-stained sections of all carcinomas were assessed for type and for histological grade, using the modified Bloom and Richardson system (Elston, 1987). Lymph node status was known for 75 cases.

Statistical analysis was by chi-square or Fisher's exact test.

### Results

*TGF-$\beta$ reactivity*

The staining of the carcinomas was classified as negative; having less than 10% positive cells; between 10 and 50% positive cells; and more than 50% positive cells. Differences were observed in the extent of reactivity with the two antibodies. The results are summarised in Table I. Staining of stroma with occasional staining of fibroblasts but without reactivity of tumour cells was only observed with the TGF-$\beta_1$ antiserum. The staining pattern with this antiserum differed in other respects, in that normal epithelium showed no staining or very weak reactivity, while tumour cell reactivity was prominent (Figure 1). This differed from the staining observed with the antibody detecting TGF-$\beta_1$ and TGF-$\beta_2$ in that normal epithelium was reactive, and the staining of tumour cells was generally of similar intensity. Of the ten carcinomas stained with both antibodies, four were negative with TGF-$\beta_1$ antibody but had 10–50% positive cells with the antibody against TGF-$\beta_1$ and TGF-$\beta_2$ (Figure 2), four

**Table I** Comparison of the extent of staining observed in breast carcinomas with the antibodies against TGF-$\beta_1$ and TGF-$\beta_1$ and -$\beta_2$

| *Reactivity* | *TGF-$\beta_1$* | *TGF-$\beta_1$ and TGF-$\beta_2$* |
|---|---|---|
| Negative | 20 (34.5%) | 3 (11%) |
| Stromal only | 7 (12%) | 0 |
| < 10% positive cells | 10 (17.25%) | 7 (25%) |
| 10–50% positive cells | 10 (17.25%) | 9 (32%) |
| > 50% positive cells | 11 (19%) | 9 (32%) |

**Figure 1** Infiltrating ductal carcinoma showing prominent staining for TGF-$\beta_1$ protein.

had less than 10% cells positive for TGF-$\beta_1$ but between 10 and 50% positive for TGF-$\beta_1$ and -$\beta_2$, and two had between 10 and 50% cells positive with TGF-$\beta_1$ antibody but more than 50% cells staining with the antibody against TGF-$\beta_1$ and TGF-$\beta_2$.

Apart from the small number of cases with stromal staining in which there was fibroblast reactivity, there was no staining of stromal cells such as macrophages.

*Relationship to clinicopathological parameters*

Seventy-five of the carcinomas were infiltrating ductal and 11 infiltrating lobular carcinomas. No differences were observed between the two categories with either antibody. There were ten well-differentiated carcinomas, 44 moderately differentiated and 32 poorly differentiated carcinomas. There was no relationship between staining and histological grade for either antibody.

Forty-five carcinomas had metastasised to lymph nodes and 30 had not. All of the carcinomas with > 50% of cells positive for TGF-$\beta_1$ had metastasised, which was significant ($0.02 > P > 0.01$). The distribution of node-positive and -negative cases for the other staining categories of TGF-$\beta_1$ was as expected, as was the distribution for all staining categories for TGF-$\beta_1$ and -$\beta_2$.

*Relationship with stromal components*

Cellular fibronectin was detected in tumour cells in 30 (35%) carcinomas (Figure 3). The extent of reactivity ranged from 10% to 80% of cells being positive, with associated lesser stromal reactivity. The other fibronectin antibody detected the stromal component, with cellular staining being seen much less frequently. The pattern of staining was predominantly of coarse bands, but in 13 carcinomas only fine irregular stromal staining was seen, and in a further 13 both coarse and fine stromal staining was observed. The comparison between fibronectin reactivity and staining for TGF-$\beta$ is shown in Table II. The presence of cellular fibronectin was greater in those cases with more prominent reactivity for TGF-$\beta_1$ and TGF-$\beta_1$ plus TGF-$\beta_2$. A greater degree of fine stromal reactivity for fibronectin was seen in cases with more prominent reactivity for TGF-$\beta_1$.

Figure 2 a, Infiltrating ductal carcinoma showing no reactivity with antibody against TGF-$\beta_1$. b, Serial section of the same carcinoma showing staining of tumour cells with antibody against TGF-$\beta_1$ and TGF-$\beta_2$.

The extent of staining for tenascin was subdivided into marked (+ + +), moderate (+ +) or scanty (+), as described previously (Jones *et al.*, 1992) (Figure 4). Marked reactivity was seen in 31 carcinomas, moderate in 29 and scanty in 11. The degree of staining in comparison with TGF-$\beta$ reactivity is shown in Table III. Marked reactivity for tenascin was seen in almost all carcinomas with prominent staining for TGF-$\beta_1$, and was significant ($0.02 > P > 0.01$), but no relationship was observed for staining with the antibody against TGF-$\beta_1$ and TGF-$\beta_2$.

*Relationship with macrophage/lymphocytic infiltration*

There was generally a greater number of cells staining in the frozen sections incubated with EBM 11 than the fixed sections reacted with PGM1, and in all cases the higher level of macrophage staining was taken for comparisons. Macrophages were seen either within the stroma or within and closely abutting tumour cell groups, subsequently called tumour associated. The extent of macrophage infiltration and whether it was stromal and/or tumour associated were related to the degree of TGF-$\beta$ reactivity within carcinomas. The extent of reactivity did not relate to TGF-$\beta$ staining. Stromal macrophage reactivity only was seen in 35 carcinomas (41%), with stromal macrophage numbers being greater than tumour associated in 16 tumours (18.5%). In 25 carcinomas (29%) there was equal reactivity for stromal and tumour-associated macrophages. Only two tumours had

Figure 3 Infiltrating ductal carcinoma with many tumour cells showing staining for cellular fibronectin.

Table II The pattern and/or extent of reactivity of stromal components in relation to the extent of staining for TGF-$\beta$ obtained with the two antibodies ($n$ = number postive/total)

| *TGF-β reactivity* | *Cellular fibronectin* | | *Coarse stromal fibronectin* | | *Fine stromal fibronectin* | |
|---|---|---|---|---|---|---|
| | *TGF-$\beta_1$* | *TGF-$\beta_1$ + TGF-$\beta_2$* | *TGF-$\beta_1$* | *TGF-$\beta_1$ + TGF-$\beta_2$* | *TGF-$\beta_1$* | *TGF-$\beta_1$ + TGF-$\beta_2$* |
| Negative | 6/20 | 0/3 | 15/20 | 1/3 | 5/20 | 1/3 |
| Stromal only | 1/7 | 0 | 4/7 | 0 | 3/7 | 0 |
| <10% | 2/10 | 0/7 | 3/10 | 4/7 | 3/10 | 1/7 |
| 10–50% | 4/10 | 3/9 | 4/10 | 7/9 | 6/10 | 2/9 |
| >50% | 8/11 | 6/9 | 4/11 | 7/9 | 6/11 | 0/9 |

**Table III** Extent of tenascin reactivity in comparison with TGF-$\beta$ staining

| *TGF-β reactivity* | *Marked tenascin* TGF-$\beta_1$ | *Marked tenascin* TGF-$\beta_1$ + -$\beta_2$ | *Moderate tenascin* TGF-$\beta_1$ | *Moderate tenascin* TGF-$\beta_1$ + -$\beta_2$ | *Scanty tenascin* TGF-$\beta_1$ | *Scanty tenascin* TGF-$\beta_1$ + -$\beta_2$ |
|---|---|---|---|---|---|---|
| Negative | 5/17 | 1/3 | 6/17 | 1/3 | 6/17 | 1/3 |
| Stromal only | 3/6 | 0 | 3/6 | 0 | 0/6 | 0 |
| <10% | 1/8 | 3/6 | 5/8 | 3/6 | 2/8 | 0/6 |
| 10–50% | 3/6 | 5/9 | 3/6 | 4/9 | 0/6 | 0/9 |
| >50% | 8/9 | 2/7 | 1/9 | 3/7 | 0/9 | 2/7 |

a b

**Figure 4** **a**, Frozen section of carcinoma showing prominent staining for tenascin. **b**, Minimal reactivity for tenascin in frozen section of another infiltrating ductal carcinoma.

more prominent tumour-associated macrophage staining, and eight carcinomas had this as the only pattern of macrophage staining. Stromal macrophage staining only or greater stromal macrophage reactivity was seen in over half the carcinomas in each TGF-$\beta$ staining category apart from those carcinomas with prominent staining for TGF-$\beta_1$ in which two showed only stromal staining, two only tumour-associated macrophage staining and the remaining seven equal reactivity for stromal and tumour-associated macrophages.

B-lymphocyte reactivity was minimal in the majority of carcinomas studied, and showed no correlation with TGF-$\beta$ reactivity. The numbers of T lymphocytes overall varied between the carcinomas, and this did not relate to TGF-$\beta$ reactivity. The extent of the CD4- and CD8-positive lymphocytes did vary and the results are shown in Table IV. Two-thirds of the carcinomas had a greater number of CD4-positive lymphocytes than CD8-positive cells, with 16% having equal numbers and 20% a greater number of CD8-positive cells. Of those carcinomas with prominent TGF-$\beta_1$ reactivity, there were two-thirds with greater CD8 reactivity.

### Discussion

In a previous immunohistochemical study of TGF-$\beta$ (Walker & Dearing, 1992) we identified a difference in detection of TGF-$\beta_1$ between *in situ* and invasive carcinomas, indicating a role for TGF-$\beta_1$ in invasion. The present study has shown that any relationship between TGF-$\beta$ and invasion and metastasis is only found for TGF-$\beta_1$ and not for TGF-$\beta_2$. This is in keeping with the findings of Gorsch *et al.* (1992), who identified a relationship between immunoreactivity for TGF-$\beta_1$ and disease progression in human breast carcinoma. It also reinforces the view of Arteaga and Coffey (1992), based on the study of McCune *et al.* (1992), that it is important to consider the different isoforms of TGF-$\beta$ since they clearly do have different roles.

Because of the availability of antisera the number of cases which could be examined for TGF-$\beta_1$ was restricted. Comparison of staining in individual cases, and of the extent of reactivity in other cases, showed that there was greater reactivity using an antibody detecting both TGF-$\beta_1$ and TGF-$\beta_2$. Because the results obtained were clearly less significant, staining with this antibody was not pursued.

Prominent reactivity for TGF-$\beta_1$ was associated with nodal metastasis, higher frequency of detection of cellular fibronectin, different patterns of reactivity of stromal fibronectin, marked tenascin reactivity, higher frequency of macrophage infiltration being tumour associated and different levels of CD8 lymphocyte infiltrates in comparison with CD4. The various correlates were not restricted to tumours with prominent TGF-$\beta_1$, and not all tumours having that pattern of TGF-$\beta_1$ reactivity showed them, but there were obvious associations. Further studies using a monospecific reagent are needed to consolidate these findings.

Studies of rat mammary adenocarcinoma cells have shown that exogenous TGF-$\beta_1$ may modulate the metastatic potential of mammary tumour cells by controlling their ability to break down and penetrate basement membrane barriers (Welch *et al.*, 1990). The TGF-$\beta_1$ secreted from tumour cells could have the same effect, providing it is biologically active. This can only be determined by *in vitro* assays. Mizoi *et al.*

**Table IV** The extent of CD4 and CD8 reactivity in carcinomas in comparison with TGF-$\beta$ staining, with both antibodies

| TGF-$\beta$ *reactivity* | CD4 > CD8 *TGF-$\beta_1$* | *TGF-$\beta_1$ + -$\beta_2$* | CD4 = CD8 *TGF-$\beta_1$* | *TGF-$\beta_1$ + -$\beta_2$* | CD8 > CD4 *TGF-$\beta_1$* | *TGF-$\beta_1$ + -$\beta_2$* |
|---|---|---|---|---|---|---|
| Negative | 13 | 2 | 2 | 0 | 3 | 1 |
| Stromal only | 3 | 0 | 3 | 0 | 0 | 0 |
| < 10% | 8 | 3 | 0 | 4 | 1 | 0 |
| 10–50% | 6 | 5 | 2 | 2 | 1 | 2 |
| > 50% | 4 | 7 | 0 | 1 | 7 | 1 |

(1993) have demonstrated in gastrointestinal carcinomas that the precursor form of TGF-$\beta_1$ is within the cytosol of tumour cells, which may suggest blocked transport. Further studies, preferably dynamic, would be required to determine whether this is the situation in breast carcinomas.

Differences in stromal and cellular fibronectin were observed relating to TGF-$\beta_1$. Previous immunohistochemical studies of stromal fibronectin have described pericellular reactivity, particularly at the invasive border, as well as a diffuse staining pattern (Christensen *et al.*, 1989). Pericellular staining was rarely seen, the more striking difference in the present study being the presence of fine stromal staining. Cytoplasmic fibronectin has previously been reported to be related to the degree of anaplasia, and more striking in independently growing breast cancer cells (Christensen *et al.*, 1985). The other extracellular matrix protein studied, tenascin, was readily identified in the stroma of the breast carcinomas, as previously reported (Mackie *et al.*, 1987; Natali *et al.*, 1991; Jones *et al.*, 1992). Tenascin is induced by TGF-$\beta$ *in vitro* (Pearson *et al.*, 1988). Tenascin can block the action of fibronectin (Chiquet-Ehrismann *et al.*, 1988), inhibiting cell attachment. *In vitro* addition of tenascin to MCF-7 breast cancer cell lines results in their loss of cell–cell and cell–substrate contacts (Chiquet-Ehrismann *et al.*, 1989). If the same occurs in primary breast carcinomas *in vivo*, it could be proposed that the overexpression of TGF-$\beta_1$ stimulates synthesis of tenascin, which aids invasion and hence metastasis.

We were unable to detect TGF-$\beta$ in macrophages within the breast carcinomas, although in other sites, such as lung, macrophages are a source of TGF-$\beta$ (Assoian *et al.*, 1987). The main findings related to the presence and relative proportion of tumour-associated macrophages, which were increased in relation to greater TGF-$\beta_1$ expression. In other tissues TGF-$\beta$ is a potent chemoattractant for macrophages. The function of macrophages within breast carcinomas could be as a host defence mechanism or the converse owing to release of enzymes involved in destruction of basement membranes, so aiding invasion. An association between nodal metastasis and macrophage infiltration has been observed (Zuk & Walker, 1987).

No differences were found in the numbers of B and T lymphocytes in relation to TGF-$\beta$ reactivity, but an alteration in the ratio of CD4 to CD8 cells was seen. As in a previous study (Zuk & Walker, 1987) CD4 lymphocytes predominated in many of the carcinomas, apart from those with prominent TGF-$\beta_1$ reactivity. Naukkarinen and Syrjanen (1990) identified an association between CD8 lymphocytic infiltration and post-capillary venule endothelium in breast carcinomas. TGF-$\beta_1$ has a role in angiogenesis and may account for this association.

Prominent expression of TGF-$\beta_1$ but not TGF-$\beta_2$ is therefore associated with changes in the extracellular matrix and in stromal infiltrates in breast carcinomas, which in view of the previously identified differences between *in situ* and invasive carcinoma and the higher frequency of nodal metastasis points to a role for TGF-$\beta_1$ in invasion and metastasis.

We are grateful to Trent Regional Health Authority for financial support and to Mrs T. Latham and Mrs M. Hornby for secretarial assistance.

### References

ARTEAGA, C.L. & COFFEY, R.J. (1992). Transforming growth factor -$\beta$ isoforms in mammary neoplasia: more questions than answers. *Hum. Pathol.*, **23,** 1–3.

ASSOIAN, R.K., FLEURDELYS, B.E., STEVENSON, H.C., MILLER, P.J., MADTES, D.K., RAINES, E.W., ROSS, R. & SPORN, M.B. (1987). Expression and secretion of type $\beta$ transforming growth factor by activated human macrophages. *Proc. Natl Acad. Sci. USA*, **84,** 6020–6024.

BARNARD, J.A., LYONS, R.H. & MOSES, H.L. (1990). The cell biology of transforming growth factor $\beta$. *Biochim. Biophys. Acta*, **1032,** 79–87.

CHANTRY, D., TURNER, M., ABNEY, E. & FELDMANN, M. (1989). Modulation of cytokine production by transforming growth factor-$\beta$. *J. Immunol.*, **142,** 4295–4300.

CHIQUET-EHRISMANN, R., KALLA, P., PEARSON, C.A., BECK, K. & CHIQUET, M. (1988). Tenascin interferes with fibronectin action. *Cell*, **53,** 383–390.

CHIQUET-EHRISMANN, R., KALLA, P. & PEARSON, C.A. (1989). Participation of tenascin and transforming growth factor-$\beta$ in reciprocal epithelial–mesenchymal interactions of MCF-7 cells and fibroblasts. *Cancer Res.*, **49,** 4322–4325.

CHRISTENSEN, L., NIELSEN, M., HOLUND, B. & CLEMMENSEN, I. (1985). In vivo demonstration of cytoplasmic fibronectin in human breast carcinomas. *Virchows Arch. (Pathol. Anat.)*, **407,** 337–346.

CHRISTENSEN, L., NIELSEN, M., ANDERSEN, J. & CLEMMENSEN, I. (1989). Stromal fibronectin staining pattern and metastasizing ability of human breast carcinoma. *Cancer Res.*, **49,** 6227–6233.

DE MARTIN, R., HAENDLER, B., HOFER-WARBINEK, R., GAUGITSCH, H., WRANN, M., SCHLUSENER, H., SEIFERT, J.M., BODMER, S., FONTANA, A. & HOFER, E. (1987). Complimentary DNA for human glioblastoma-derived T cell suppressor factor, a novel member of the transforming growth factor-$\beta$ gene family. *EMBO J.*, **6,** 3673–3677.

DERYNCK, R., JARRETT, J.A., CHEN, E.Y., EATON, D.H., BELL, J.R., ASSOIAN, R.K., ROBERTS, A.B., SPORN, M.B. & GOEDDEL, D.V. (1985). Human transforming growth factor-$\beta$ complementary DNA sequence and expression in normal and transformed cells. *Nature*, **316,** 701–705.

DERYNCK, R., LINDQUIST, P.B., LEE, A., WEN, D., TAMM, J., GRAYCAR, J.L., RHEE, L., MASON, A.J., MILLER, D.A., COFFEY, R.J., MOSES, H.L. & CHEN, E.Y. (1988). A new type of transforming growth factor-$\beta$, TGF-$\beta$3. *EMBO J.*, **7,** 3737–3743.

EDWARDS, D.R., MURPHY, G., REYNOLDS, J.J., WHITHAM, S.E., DOCHERTY, J., ANGEL, P. & HEATH, J.C. (1987). Transforming growth factor beta modulates the expression of collagenase and metalloproteinase inhibitor. *EMBO J.*, **6,** 1899–1904.

ELSTON, C.W. (1987). Grading of invasive carcinoma of the breast. In *Diagnostic Histopathology of the Breast*, Page, D.L. & Anderson, T.J. (eds) pp. 300–311. Churchill Livingstone: Edinburgh.

GORSCH, S.M., MEMOLI, V.A., STUKEL, T.A., GOLD, L.I. & ARRICK, B.A. (1992). Immunohistochemical staining for transforming growth factor $\beta$ associates with disease progression in human breast cancer. *Cancer Res.*, **52,** 6949–6952.

IGNOTZ, R.A. & MASSAGUE, J. (1986). Transforming growth factor-$\beta$ stimulates the expression of fibronectin and collagen and their incorporation into the extracellular matrix. *J. Biol. Chem.*, **261**, 4337–4345.

JAKOWLEW, S.B., DILLARD, P.J., SPORN, M.B. & ROBERTS, A.B. (1988). Complementary deoxyribonucleic acid cloning of an mRNA encoding transforming growth factor-beta 4 from chicken embryo chondrocytes. *Mol. Endocrinol.*, **2**, 1186–1195.

JONES, J.L., CRITCHLEY, D.R. & WALKER, R.A. (1992). Alteration of stromal protein and integrin expression in breast – a marker of premalignant change? *J. Pathol.*, **167**, 399–406.

KEHRL, J.H., WAKEFIELD, L.M., ROBERTS, A.B., JACOWLEW, S., ALVAREZ-MON, M., DERYNCK, R.M., SPORN, M.B. & FAUCI, A.S. (1986*a*). Production of transforming growth factor $\beta$ by human T lymphocytes and its potential role in the regulation of T cell growth. *J. Exp. Med.*, **163**, 1037–1050.

KEHRL, J.H., ROBERTS, A.B., WAKEFIELD, L.M., JAKOWLEW, S., SPORN, M.B. & FAUCI, A.S. (1986*b*). Transforming growth factor $\beta$ is an important immunomodulatory protein for human B lymphocytes. *J. Immunol.*, **137**, 3855–3860.

KONDAIAH, P., SANDS, M.J., SMITH, J.M., FIELDS, A., ROBERTS, A.B., SPORN, M.B. & MELTON, D.A. (1990). Identification of a novel transforming growth factor-$\beta$ (TGF-$\beta$5) mRNA in *Xenopus laevis*. *J. Biol. Chem.*, **265**, 1089–1093.

MCCUNE, B.K., MULLIN, B.R., FLANDERS, K.C., JAFFURS, W.J., MULLEN, L.T. & SPORN, M.B. (1992). Localization of transforming growth factor-$\beta$ isotypes in lesions of the human breast. *Hum. Pathol.*, **23**, 13–20.

MACKIE, R.J., CHIQUET-EHRISMANN, R., PEARSON, C.A., INAGUMA, J., TAYA, K., KAWARADA, J. & SAKAKURA, T. (1987). Tenascin is a stromal marker for epithelial malignancy in the mammary gland. *Proc. Natl Acad. Sci. USA*, **84**, 4621–4625.

MIZOI, T., OHTANI, H., MIYAZANO, K., MIYAZAWA, M., MATSUNO, S. & NAGURA, H. (1993). Immunoelectron microscopic localization of transforming growth factor $\beta_1$ and latent transforming growth factor $\beta_1$ binding protein in human gastrointestinal carcinomas: qualitative difference between cancer cells and stromal cells. *Cancer Res.*, **53**, 183–190.

NATALI, P.G., NICOTRA, M.R., BIGOTTI, A., BOTTI, C., CASTELLANI, P., RISSO, A.M. & ZARDI, I. (1991). Comparative analysis of the expression of the extracellular matrix protein tenascin in normal human fetal adult and tumour tissues. *Int. J. Cancer*, **47**, 811–816.

NAUKKARINEN, A. & SYRJANEN, K.J. (1990). Quantitative immunohistochemical analysis of mononuclear infiltrates in breast carcinomas – correlation with tumour differentiation. *J. Pathol.*, **160**, 217–222.

PEARSON, C.A., PEARSON, D., SHIBAHARA, S., HOFSTEENGE, J. & CHIQUET-EHRISMANN, R. (1988). Tenascin: cDNA cloning and induction by TGF-$\beta$. *EMBO J.*, **7**, 2977–2981.

ROBERTS, A.B., THOMPSON, N.L., HEINE, U., FLANDERS, K. & SPORN, M.B. (1988). Transforming growth factor beta: possible roles in carcinogenesis. *Br. J. Cancer*, **57**, 594–600.

TSUNAWAKI, S., SPORN, M., DING, A. & NATHAN, C. (1988). Deactivation of macrophages by transforming growth factor-$\beta$. *Nature*, **334**, 260–262.

VAN DER HOOFF, A. (1988). Stromal involvement in malignant growth. *Adv. Cancer Res.*, **50**, 159–196.

VOSE, B.M. & MOORE, M. (1985). Human tumour-infiltrating lymphocytes: a marker of host response. *Semin. Haematol.*, **22**, 27–40.

WALKER, R.A. & DEARING, S.J. (1992). Transforming growth factor beta, in ductal carcinoma in situ and invasive carcinomas of the breast. *Eur. J. Cancer*, **28**, 641–644.

WELCH, D.R., FABRA, A. & NAKAJIMA, M. (1990). Transforming growth factor $\beta$ stimulates mammary adenocarcinoma cell invasion and metastatic potential. *Proc. Natl Acad. Sci. USA*, **87**, 7678–7682.

ZUK, J.A. & WALKER, R.A. (1987). Immunohistochemical analysis of HLA antigens and mononuclear infiltrates of benign and malignant breast. *J. Pathol.*, **152**, 278–285.